# Oxygenation improves during the first 8 h of extended-duration prone positioning in patients with respiratory failure: a retrospective study

**DOI:** 10.1186/s40560-014-0052-5

**Published:** 2014-09-05

**Authors:** Kyohei Miyamoto, Yu Kawazoe, Masato Yasuda, Naoaki Shibata, Tsuyoshi Nakashima, Maki Kida, Seiya Kato

**Affiliations:** Department of Emergency and Critical Care Medicine, Wakayama Medical University, Wakayama, 641-8509 Japan

**Keywords:** Prone positioning, Respiratory failure, Oxygenation

## Abstract

**Background:**

A recent multicenter trial demonstrated decreased mortality when patients with acute respiratory distress syndrome were treated with prone positioning (PP). However, the optimal duration of this treatment has not been established.

**Methods:**

From May 2010 to August 2013, 15 patients with respiratory failure underwent extended-duration prone positioning (more than 40 h) in the medical-surgical intensive care unit of a university hospital. The records of each patient were retrospectively investigated to evaluate the impact of prone positioning on the PaO_2_/FiO_2_ ratio (PFR) during the first 40 h of therapy.

**Results:**

The mean age of the patients was 72.2 ± 7.8 years, and the mean Acute Physiology and Chronic Health Evaluation II score was 19.0 ± 6.0. The hospital mortality rate was 47% (7/15), and the median duration of prone positioning was 47.5 h (46.5–67). The mean PFR before prone positioning was 193.8 ± 70.1, and it significantly improved to 274.7 ± 70.7 (*p* = 0.02) at 8 h after prone positioning initiation. Although PFR further improved to 294.1 ± 78.0 (*p* = 0.23) at 16 h, the change was not significant and PFR remained relatively constant at 289.0 ± 88.1, 294.6 ± 68.2, and 291.7 ± 72.7 at 24, 32, and 40 h, respectively.

**Conclusions:**

Extended-duration prone positioning resulted in a progressive improvement in oxygenation until 8 to 16 h after treatment initiation, after which there was no significant improvement. Further studies are warranted to clarify the optimal duration of prone positioning and the actual effectiveness of extended-duration PP for respiratory failure.

## Background

Acute respiratory distress syndrome (ARDS) has various etiologies and poses a therapeutic challenge. In the past decades, many studies demonstrated that prone positioning (PP) improved oxygenation in ARDS patients [1–4]. Moreover, PP has increasingly attracted attention since it was reported to decrease the mortality rate in patients with severe ARDS in the Proning Severe ARDS Patients (PROSEVA) trial in 2013 [5]. However, the optimal method of performing PP has not been established. In particular, the duration of PP varies from 6 to 20 h [1–5] daily, and few studies examined the effects of >24 h PP on oxygenation.

We generally perform PP in patients with refractory ARDS or weaning failure who fail to respond to standard ventilatory support. We performed extended-duration PP for the reversal of atelectasis for successive 48–72 h in a patient with refractory respiratory failure. In this study, we examined the effects of extended-duration PP on oxygenation in patients with respiratory failure.

## Methods

We retrospectively analyzed the medical records of 15 patients with respiratory failure who underwent extended-duration PP (15 episodes) in the medical-surgical intensive care unit (ICU) at Wakayama Medical University, which has ten beds and a closed-ICU system, from May 2010 to August 2013. Extended-duration PP was defined as PP lasting for ≥40 h. Patients who underwent PP for <40 h were excluded.

ARDS was diagnosed and classified according to the Berlin definition [6]. Briefly, mild ARDS was defined by a PaO_2_/FiO_2_ ratio (PFR) of >200, moderate ARDS by a PFR of 100–200, and severe ARDS by a PFR of <100.

The data evaluated included the findings of blood gas analysis, ventilator settings, and circulatory index before PP; at 8, 16, 24, 32, and 40 h after PP initiation; and at 8, 16, 24, 32, and 40 h after PP completion. We used the blood gas data obtained at the nearest time from each time point. Any complications related to PP were confirmed from the nurses’ notes on the medical charts of patients. This study was approved by the institutional review board at Wakayama Medical University, which waived the requirement for informed consent because of the observational nature of the study.

### Statistical analysis

Continuous variables are presented as the mean ± standard deviation (SD) or the median (interquartile range). Categorical variables are presented as numbers and percentages (%). Analysis of variance (ANOVA) was used for comparisons among the various time points. When significant differences were detected, additional multiple comparisons were performed using Dunnett’s test with baseline values (before PP, supine position) as reference values. Paired *t*-tests were also used for comparisons of values before and after PP. Statistical analyses were performed using JMP version 9.0 (SAS Institute, Cary, NC, USA). The *p* value <0.05 was considered statistically significant.

## Results

From May 2010 to August 2013, we performed 20 episodes of PP in 19 patients. Five episodes were interrupted before the completion of planned extended-duration PP and were excluded from analysis. The reasons for interruption were arrhythmia (*n* = 2), hypotension (*n* = 1), requirement of central venous catheter insertion (*n* = 1), and unknown (*n* = 1). Eventually, 15 episodes in 15 patients were analyzed.

The characteristics of the patients are shown in Table 1. The mean age of the patients was 72.2 ± 7.8 years. The mean Acute Physiology and Chronic Health Evaluation (APACHE) II score on admission to the ICU was 19.0 ± 6.0. The causes of respiratory failure included sepsis (*n* = 12; including seven cases of pneumonia), near drowning (*n* = 1), burns (*n* = 1), and atelectasis (*n* = 1). We diagnosed ARDS in 12 patients (80%), 11 of whom had moderate or severe ARDS. The hospital mortality rate was 47% (*n* = 7). The median duration of PP was 47.5 h (46.5–67).

**Table 1:** **Clinical characteristics of patients**

	**PP (** ***n*** **= 15)**
Age, years	72.2 ± 7.8
Men, *n* (%)	11 (73%)
APACHE II score	19.0 ± 6.0
Cause of respiratory failure	
Sepsis with pneumonia, *n* (%)	7 (47%)
Sepsis without pneumonia, *n* (%)	5 (33%)
Others, *n* (%)	3 (20%)
Severity of ARDS during ICU stay	
Not ARDS, *n* (%)	3 (20%)
Mild, *n* (%)	1 (7%)
Moderate, *n* (%)	7 (46%)
Severe, *n* (%)	4 (27%)
Treatment in ICU	
Vasopressor, *n* (%)	11 (73%)
RRT, *n* (%)	3 (20%)
Mode of MV during PV	
APRV, *n* (%)	6 (40%)
SIMV, *n* (%)	4 (27%)
A/C, *n* (%)	3 (20%)
CPAP/PSV, *n* (%)	2 (13%)
Duration of PP (h): median (IQR)	47.5 (46.5–67)
MV before starting PP (days): median (IQR)	3 (2–6)
Hospital mortality, *n* (%)	7 (47%)

Values are expressed as the mean ± standard deviation, unless otherwise indicated. APACHE II score was calculated at the admission to ICU. *PP* prone positioning, *APACHE II* Acute Physiology and Chronic Health Evaluation II, *ARDS* acute respiratory distress syndrome, *ICU* intensive care unit, *RRT* renal replacement therapy, *MV* mechanical ventilation, *APRV* airway pressure releasing ventilation, *SIMV* synchronized intermittent mandatory ventilation, *A*/*C* assist/control, *CPAP* continuous positive airway pressure, *PSV* pressure support ventilation, *IQR* interquartile range.

Blood gas analysis was performed after starting PP at 486 ± 145 min (8 h), 997 ± 113 min (16 h), 1,446 ± 117 min (24 h), 1,976 ± 148 min (32 h), and 2,430 ± 140 min (40 h).

**Figure 1:**
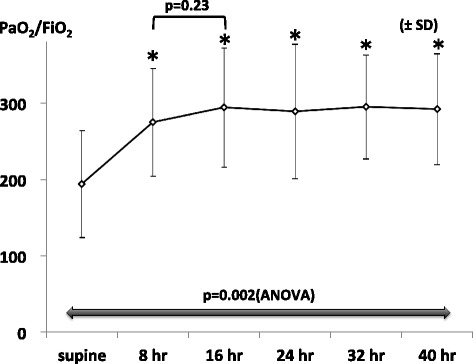
**Time course of changes in the PaO**_**2**_ **/FiO**_**2**_ **ratio (PFR) during the first 40 h of prone positioning (PP).** Analysis of variance (ANOVA) was used to compare values among the various time points. Multiple comparisons were performed using Dunnett’s tests with baseline values (before PP; supine position) as reference values. The paired *t*-test was also used to compare the values at 8 h with those at 16 h. *Supine* represents immediately before starting prone positioning (*p < 0.05 vs. reference). *SD* standard deviation.

The time course of the changes in PFR is shown in Figure 1. Compared with the mean PFR before PP (193.8 ± 70.1), we identified a significant improvement at 8 (274.7 ± 70.7; *p* = 0.02), 16 (294.1 ± 78.0; *p* = 0.002), 24 (289.0 ± 88.1; *p* = 0.002), 32 (294.6 ± 68.2; *p* = 0.002), and 40 h (291.7 ± 72.7; *p* = 0.003) after PP initiation. Compared with the mean PFR at 8 h, however, the improvement at 16 h was not significant (*p* = 0.23) and remained relatively constant up to 40 h.

The time course of changes in other ventilation and hemodynamic variables is shown in Table 2. There was no significant change in PEEP during the course of PP (*p* = 0.99).

**Table 2:** **Ventilation, oxygenation, and hemodynamic variables throughout the first 40 h of the prone positioning**

	**Supine**	**8 h**	**16 h**	**24 h**	**32 h**	**40 h**	***p*** **value**
HR	89.7 ± 18.2	88.8 ± 20.3	85.6 ± 20.0	89.7 ± 19.0	89.3 ± 22.5	84.3 ± 17.2	0.95
MBP	78.9 ± 14.7	80.6 ± 10.1	77.4 ± 14.4	75.8 ± 14.5	80.1 ± 19.2	75.5 ± 13.9	0.90
FiO_2_	0.62 ± 0.19	0.50 ± 0.13	0.51 ± 0.12	0.48 ± 0.09	0.46 ± 0.09	0.46 ± 0.09	0.002
PEEP	13.4 ± 6.9	13.5 ± 6.4	13.5 ± 6.4	13.9 ± 6.5	12.9 ± 6.5	12.4 ± 6.0	0.99
PFR	193.8 ± 70.1	274.7 ± 70.7	294.1 ± 78.0	289.0 ± 88.1	294.6 ± 68.2	291.7 ± 72.7	0.002
PaCO_2_	43.8 ± 7.7	47.5 ± 8.0	46.0 ± 13.6	44.9 ± 10.6	46.3 ± 8.9	47.2 ± 8.5	0.91

Values are expressed as the mean ± standard deviation. ANOVA was used to compare values among the various time points. Supine represents immediately before starting prone positioning. *HR* heart rate, *MBP* mean blood pressure, *PEEP* positive end-expiratory pressure, *PFR* PaO_2_/FiO_2_ ratio.

**Figure 2:**
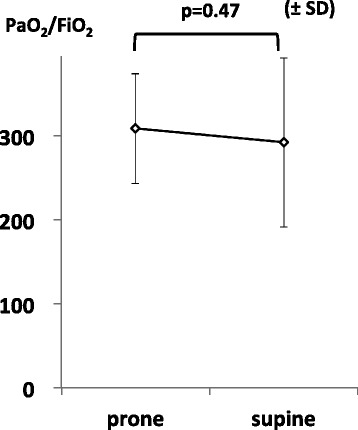
**Mean PFR before and after PP.** Paired *t*-tests were used to compare values among the various time points. *Prone* represents immediately before completing prone positioning. *Supine* represents 8 h after completing prone positioning. *SD* standard deviation.

After PP was completed, blood gas analysis was performed at 507 ± 128 min. Compared with the mean PFR before PP completion, there was no significant decrease at 8 h after PP completion (308.4 ± 65.4 vs. 292.1 ± 100.8, respectively; *p* = 0.47; Figure 2).

Within 40 h after PP completion, five patients were extubated and one patient died. In the other nine patients, blood gas analysis was performed after completion of PP at 507 ± 123 min (8 h), 1,076 ± 152 min (16 h), 1,497 ± 140 min (24 h), 1,960 ± 128 min (32 h), and 2,485 ± 141 min (40 h). In these patients, the PFR was 308.3 ± 63.4 before PP completion and 300.2 ± 97.1 at 8 h after PP completion, 274.0 ± 80.1 at 16 h, 253.4 ± 84.2 at 24 h, 260.0 ± 87.4 at 32 h, and 270.5 ± 105.0 at 40 h. There was no significant change in PFR within 40 h after completion of PP (*p* = 0.72).

Three patients developed a mild facial pressure ulcer (partial-thickness loss of dermis) during PP. Unintended extubation or displacement of lines did not occur in any patient. A patient developed bradycardia, and another patient developed oliguria during PP.

## Discussion

In this study, we examined the effects of extended-duration PP (median duration, 47.5 h) on oxygenation in patients with respiratory failure. Although we identified a significant improvement in PFR at 8 h after PP initiation, there was no significant improvement thereafter.

To date, several studies have examined the effectiveness of PP in the treatment of ARDS. In 2001, Gattinoni et al. conducted the first randomized controlled trial (RCT) of short-duration PP (6 h daily) for ARDS [1]. They concluded that PP did not improve the survival of ARDS patients; however, a survival benefit was evident in the severe ARDS subgroup (PFR <88). Following this trial, several studies on longer-duration PP for more severe ARDS were conducted. In 2013, the PROSEVA trial showed a significant decrease in 28- and 90-day mortality rates after longer-duration PP for severe ARDS [5]. This RCT enrolled patients with severe ARDS, who exhibited a PFR of <150 after standard ventilatory management for >12 h. The minimum duration of PP in this trial was 16 h daily.

The method of PP varies considerably among institutions. In particular, the optimal duration of PP has not been established. A recent meta-analysis suggested that the outcomes of PP were better in a subgroup that received the treatment for a longer duration (≥10 h daily) [7], which resulted in a significant decrease in mortality rate (odds ratio, 0.62; 95% confidence interval, 0.48–0.79). In contrast, the mortality rate was not decreased in the subgroup that received a shorter duration of PP (<10 h daily; odds ratio, 1.04; 95% confidence interval, 0.80–1.36).

Although many studies have examined the effect of PP lasting for 16–20 h daily [3–5], few have examined the effects of PP lasting for >24 h. A prospective observational study examined the effects of extended-duration PP (average, 55 h) in 15 patients with severe ARDS and demonstrated a gradual improvement in oxygenation during the course of PP [8]. However, the detailed time course of changes in oxygenation was not examined in this study.

In our study, we confirmed a significant improvement in oxygenation 8 h after PP initiation and a further nonsignificant improvement at 16 h, following which the PFR values remained relatively constant up to 40 h.

Furthermore, PEEP remained unchanged during extended-duration PP, indicating that there was no advantage of extended-duration PP from the perspective of weaning from ventilation.

Although past studies have confirmed a decrease in oxygenation after PP completion [1,9], there was no such decrease in the present study. This finding was supported by the abovementioned prospective observational study, which also did not observe a decrease in oxygenation after the completion of extended-duration PP [8]. These findings indicate that extended-duration PP may prevent the decrease in oxygenation after PP completion.

The strength of our study is that it evaluated the detailed time course of changes in oxygenation during extended-duration PP. The findings provide valuable information that can aid in determining the optimal duration of the rescue PP for refractory hypoxemia and in evaluating the response to PP in an individual patient.

Our study also has several limitations. First, it was based on a retrospective observational design without a control group. Therefore, it is difficult to determine whether the improved oxygenation was the effect of PP or the result of a natural course. In the same way, the maintained oxygenation after completion of extended-duration PP may be the result of a spontaneous recovery. Second, our study did not examine the preventive effects of PP against ventilator-induced lung injury (VILI). The mechanism underlying the improvement in outcome after PP is suggested to be protection against VILI and improved oxygenation [10]. But a recent *post hoc* analysis of findings from the PROSEVA trial suggested that PP-induced improvements in gas exchange did not predict improved outcomes [11]. The improved oxygenation may not be used as a surrogate for an improved outcome. Third, the severity of patients in our study was highly variable because there was no established protocol for the implementation of PP in our institute. Recent trials mainly targeted moderate and severe ARDS [3–5]. We may not be able to apply our results to severe ARDS patients.

Further studies are required to examine the effects of extended-duration PP using established criteria and to evaluate mortality rates and the duration of mechanical ventilation, in addition to oxygenation.

## Conclusions

PP is useful for improving oxygenation in patients with respiratory failure, as evidenced by the significant improvement in oxygenation during the first 8–16 h after PP initiation. However, there was no significant improvement after this period. Further studies are warranted to identify the optimal duration of PP for patients with respiratory failure and determine the actual effectiveness of extended-duration PP.

### Availability of supporting data

The data sets supporting the results of this article are included within the article.

## Abbreviations

PP: Prone positioning
PFR: PaO_2_/FiO_2_ ratio
PaO_2_: Partial pressure of arterial oxygen tension
FiO_2_: Inspiratory oxygen fraction
APACHE II: Acute Physiology and Chronic Health Evaluation II
ARDS: Acute respiratory distress syndrome
PROSEVA trial: The Proning Severe ARDS Patients trial
ICU: Intensive care unit
SD: Standard deviation
ANOVA: Analysis of variance
PEEP: positive end-expiratory pressure
RCT: Randomized controlled trial
VILI: Ventilator-induced lung injury
MV: Mechanical ventilation
APRV: Airway pressure releasing ventilation
SIMV: Synchronized intermittent mandatory ventilation
A/C: Assist/control
CPAP: Continuous positive airway pressure
PSV: Pressure support ventilation
RRT: Renal replacement therapy
